# The New York Institute of Stomatology

**Published:** 1897-06

**Authors:** 

**Affiliations:** The New York Institute of Stomatology


### Reports of Society Meetings.

### THE NEW YORK INSTITUTE OF STOMATOLOGY.

A REGULAR meeting of the Institute was held Tuesday evening, February 2, 1897, at the office of Dr. W. St. George Elliott, No. 25 East Forty-fourth Street, New York City, the President, Dr. George S. Allan, in the chair.

The secretary, Dr. W. St. George Elliott, read the minutes of the previous meeting, which were approved.

COMMUNICATIONS ON THEORY AND PRACTICE.

*Dr. George A. Maxfield.*—I have been very much interested in the articles of Dr. Black on amalgam, especially the last one in the December number of the *Dental Cosmos.* As I have made my own alloys for the last thirteen years, I made some from the formula which, according to his tables, gave the best results, and I brought some of the cuttings to-night, that all may see the benefit of his method of annealing the alloy. When amalgam is first cut it sets very quickly. I have two samples here which were cut yesterday, one annealed and the other not. In this last article Dr. Black has endeavored to present methods by which manufacturers will be able to furnish a good and reliable article. He has shown by experiments the shrinkage and expansion of the different alloys, and to get an alloy whose shrinkage or expansion is very slight, and at the same time one which will keep an indefinite time without change, is the object desired. I made a filling of this freshly cut amalgam yesterday afternoon, and I hardly had time to finish it, it set so quickly. After the patient had gone, I mixed some more, and also mixed some that was tempered, comparing the two, and I found the tempered alloy could be worked about five minutes after it was mixed. The process of tempering is very simple, the alloy being kept in boiling water for fifteen minutes. Dr. Flagg, in his book on plastics and plastic fillings, speaks of it as "aging," and says if it can be kept in constant motion for some time it gives it that quality. Dr. Black tested this by placing the alloy on a machine in a woollen-mill, where it had a constant reciprocating motion, and, after leaving it there for some time, found that no effect had been produced on the alloy.

This formula is five per cent. copper, sixty-one and three-fourths per cent. silver, and thirty-three and one-fourth per cent. tin. This seemed to stand the test the best of all the formulæ he tried. There is still one great trouble that will have to be overcome, and that is in regard to melting the alloys. There is more or less loss in melting. Dr. Black has averaged about one-half of one per cent., yet he had one case where it was nearly three per cent.,—twenty-six grains out of eight hundred. I saw a statement, supposed to have been written by Dr. Lawrence some years ago, in which he said he always had a loss. It is not always possible to know what the loss is. Dr. Black said he supposed it was tin. In the lot in which he had the heaviest per cent. loss, he could not tell what caused it.

*Dr. Bogue.*—He did not assay after meeting the loss?

*Dr. Maxfield.*—It simply weighed less. The specific gravity was the same.

*Dr. Bogue.*—But he could have made a quantitative analysis.

*Dr. Maxfield.*—I suppose he could, but he said he had not been able to as yet. To me this simple fact of tempering the alloys is worth a great deal.

*Dr. Bogue.*—Does Dr. Maxfield put the alloy loose in the water?

*Dr. Maxfield.*—No; I put it in a little bottle such as gold cylinders come in. Dr. Black puts his in a flask. Dr. Black says the quicker an alloy can be used after it is cut the better the results. It is not always convenient for us to stop and file the alloy as we want it. Many who do not make the alloys must buy them, and they want an amalgam that does not shrink or expand. It would be impossible with the alloy I first passed around to make a large filling or one of difficult access; but with the second lot one could fill several cavities before it sets. This alloy, before it is annealed, expands but does not shrink; but afterwards it shrinks five points.

*Dr. Elliott.*—The subject of amalgam is, perhaps, one of the most interesting we have at the present time. It is, however, wrapped up in a great deal of obscurity. I worked at it for a number of months some years ago, and arrived at certain conclusions which have in a measure been corroborated by Dr. Black, who deserves a great deal of credit for what he has done. He has discovered the true function of so-called "aging." The great trouble with us dentists is that we make up our minds that a thing is so, and state that it is so, but fail to offer any proof of our position. Dr. Black has made some variation in this matter, and has attempted to substantiate the statements which he has made. I read before the Odontological Society of London, I think in 1888, quite an extensive paper on amalgam. I tested over fifty amalgams for shrinkage, expansion, and edge-strength, but did not get a single real example of expansion. The principle I used—the specific gravity test—was not original, for it had formerly been used by Mr. Charles Tomes. I consider it preferable to the mechanical, which test must be imperfect, in my judgment. In the first place, the specific gravity test takes into consideration every part of the surface. The measurement system measures the changes in one or two directions only. One peculiar feature which I noticed, and which finally made me so dissatisfied with the investigation that I threw it up, was the uncertainty in regard to the materials. Mayhew & Co., supplied me, and they are supposed to furnish the best in London, but they could not assure me that the metals were absolutely pure. Fletcher, of Warrington, says there may be a difference of ten per cent. sometimes between different parts of the same ingot. Amalgam seems to be always undergoing some change. I kept up the tests for three months, and there was more or less change all the time. I have an apparatus here for making new experiments. The vessel contains lukewarm water of the temperature of 100° F. The lumps of amalgam, about the size of large fillings, are put in and are kept in water during the time of the experiments, the heat being kept constant by this thermostat. We all know from a practical point of view that there are hardly any two amalgams which cut alike. Some are very hard, and some can be cut through very easily. Some, by test, were four times as strong as others. As yet we are very much in the dark. I do not think the antiseptic properties of amalgams are of much importance if we can have a reliable non-shrinkage metal. The antiseptic property was a necessity in amalgams that shrank, but the ideal alloy is one that while it does not shrink, yet does possess antiseptic properties, so that in case of a recurrence of decay, this property may prove useful. It should also keep a good color. Whether it is possible to make this happy combination I doubt, for the antiseptic property seems to be associated with the color, and to be in the ratio of its depth, but this is generally owing to the use of copper in the alloy, and might be avoided by substituting a metal having a white oxide, and yet possessed of the necessary tooth-saving property.

*The President.*—I now have the pleasure of asking you to listen to Dr. Fred A. Peeso, of Philadelphia. A year or so ago I had the great pleasure of seeing a piece of practical bridge-work made by Dr. Peeso, and it impressed me so strongly as being the most finished piece of work that I had ever seen, so beautifully adapted, so practical and so clean, and did away with so many of the objections that are inherent with bridge-work, that it has been constantly on my mind to bring him to New York and have him not only show us some of the practical pieces that have been in the mouth, but also to explain to us the principle on which he constructs his work, and the tools he works with.

The doctor has brought with him some cases that have been in practical use for some time. These are valuable, and at the same time very instructive, and I am exceedingly anxious that every one should see them. I will ask Dr. Peeso to [illegible] forward and have a stand in front of him with the cases on it, so all can get near and see them. So far as I know, these specimens are perfect in all details. They are not show-pieces. They were made for patients in want of them, and have been and are now being worn with comfort and satisfaction. We are greatly indebted to Dr. Peeso for bringing them on, and to his patients for allowing him to do so.

(For Dr. Peeso's paper, see page 285.)

*Dr. Peeso.*—While I was writing this paper I saw an article in one of the journals stating that if a bridge lasted three or four years it had done all that could be expected of it, and the dentist had fulfilled his obligation to his patients. If bridge-work would last no longer than that it would certainly be a complete failure, and should not be recognized by the profession.

These cases are all practical, having been in the mouth for longer or shorter periods, most of them being extreme cases of bridge-work, and many of them having but one anchorage. They were started as an experiment, but in every case they have proved most satisfactory. Until I see some ill results following their use, I feel that I am justified in adhering to the methods which I have found so useful. These pieces are not plated, as has been suggested by some one, but are polished in the manner described.

Dr. Peeso then exhibited the pieces he had brought with him, and explained the same.

The secretary then read a paper by Dr. Adam Flickinger, of St. Louis, Missouri, on "Removable Porcelain Crown- and Bridge-work."

(For Dr. Flickinger's paper, see page 306.)

DISCUSSION.

*The President.*—Gentlemen, these two papers are now before you for discussion, and, if you please, we will hear first from Dr. George Evans.

*Dr. George Evans.*—What we have heard this evening indicates Dr. Peeso to be an honest and enthusiastic worker in the specialty of crown- and bridge-work.

I fully agree with Dr. Peeso in his introductory remarks on the subject. The usefulness of the profession to the public depends on its art. Judged from that stand-point, crown- and bridge-work is one of the most important branches practised at the present time. Most practising dentists are gradually becoming convinced that it is a branch which can no longer be ignored, and that knowledge of it is a necessary requirement.

Properly practised crown- and bridge-work approaches a fine art, but misapprehension of the principles underlying it and lack of judgment and skill in their application have conspired to mar the value of this most valuable branch of dental prosthesis.

Surgical and mechanical operations of the most delicate nature are involved in crown- and bridge-work. Nothing in dentistry demands finer manipulation. There is no branch in which experience tends more to contribute to successful results, and a high position in dental art should be accorded it. Those who are interested in its advancement grow more confident of the value of crown- and bridge-work and its principles in conservative practice. They also appreciate the fact that an elevated position in dental prosthesis has been acquired for it, as it has been added to the curriculum of every dental college in the United States as an advanced branch of education. I agree with Dr. Peeso regarding the abuse of bridge-work. Its value entirely depends on conditions being favorable to its insertion, and then skilful construction. Either lacking, it is the worst form of denture the patient can have, in fact, a misfortune. This is the reason that so much injury is done to persons patronizing fraudulent dental offices, and the good name of crown- and bridge-work is blurred.

Regarding the methods of construction of removable bridge-work as presented by Dr. Peeso, they show a careful method of procedure. They are those most generally used for that style of work with some novelties introduced.

In the trimming and shaping of natural crowns and roots I find corundum paper disks and strips a useful adjunct in smoothing the cervices and sides. The great objection to removable bridge-work is the intricacy and labor of construction. In my own practice I almost entirely limit its use to the lower jaw. My present practice of using a combination of oxyphosphate and gutta-percha for cementing makes the removal of a fixed bridge for repair or other purpose an easy matter. I will say, since I have seen Dr. Peeso's work, that it is in finish and construction equal, if not superior, to anything of its style that I have ever seen.

*The President.*—Will Dr. Evans please explain his method of using gutta-percha and cement together, so the permanent pieces can be easily removed?

*Dr. Evans.*—Take, for instance, a single collar crown with a a post. I believe in good long posts, as stated by Dr. Peeso. Having everything ready to put on, I dry the parts, using the hot-air syringe. I heat the crown as hot as I can safely without fracturing the porcelain, and then paint the post with chloropercha. The heat quickly evaporates the chloroform, leaving the gutta-percha. When the crown chills a little, I insert it in position, and as I do that the gutta-percha is moulded or scraped to the sides of the post. I do not wait for the crown to cool, but instantly remove it. I then scrape from around the base of the post in the cap any surplus that may be there. When the crown has cooled, I replace it on the root and see that it goes into position. I then cement the crown on with oxyphosphate, placing the cement in the root-canal, the same as if no gutta-percha had been placed on the post. If it is attached to a bridge, to remove it I take a short-pointed root-canal dryer, and heat the bulb portion nearly red-hot. I protect the lips of the patient with a napkin and apply the silver point of the dryer to the metallic part of the crown, telling my patient to raise the left hand when he feels the heat. When the signal comes I remove the instrument and then apply it again and continue to do so for intervals of a few moments at a time until the crown and post are uniformly heated. This heat gradually softens the sheath of gutta-percha around the post, and with moderate force the crown can be removed. I will describe briefly how I would cement a molar cap, a portion of the natural tooth having been lost. On that portion that would represent the vacuum inside the cap I put my gutta-percha in position. I warm the crown as I do this, and insert it in position in the mouth. I instantly remove the crown, and to the gutta-percha I have already placed inside the cap I add a film around on the sides. I place the crown in position and remove several times, heating and adding more gutta-percha until I have just about enough to hold it on the tooth. I then remove the crown and scrape from around the edges of the inside all that comes near the edge of the cap. Then I cement the crown on with phosphate as usual. To prevent the gutta-percha from adhering to the surface of the natural crown in the fitting, I paint it over with oil of cloves and wipe it nearly dry with absorbent cotton. The oil should be removed when ready to cement the crown.

To remove a crown cemented in this manner, heat should be applied to the grinding surface gradually, and, as a rule, the crown can be removed. There are some short crowns in which this method will not work, and in those cases use the oxyphosphate cement alone. To remove such a crown, drill a hole in the centre of the occluding surface, put in an instrument, and break the attachment, slipping off the crown. The hole can be filled with gold or amalgam when recemented. When caps on the abutments of a bridge have been cemented on with this combination of gutta-percha and oxyphosphate, they can be easily removed.

*The President.*—We would be pleased to hear from Dr. Northrop.

*Dr. H. W. Northrop.*—This is an embarrassing position for me to be placed in, and I give Dr. Woodward, who enticed me here, all the blame. In a laboratory, alone, with my coat off, I feel at home; but before these gentlemen, as an orator, I fear I shall be unable to acquit myself creditably. I am not here to oppose bridge-work, but as one of its strongest advocates. There are so many different branches of bridge-work, each having its respective merits, that I do not feel justified in criticising any one of them, although some methods I prefer to others.

The paper by Dr. Flickinger, just read, applies more to the porcelain system of bridge-work, a branch in which I have had limited experience, as my work is mostly of the kind constructed of gold, which I advocate for durability and service.

Dr. Flickinger also dwells upon removable bridges, another feature which I have had little occasion to adopt in preference to cemented work, and only in cases where the latter could not be used. The successful results which I have attained fully uphold me in expressing myself so strongly upon these two points.

There are several objections to the methods practised by Dr. Flickinger I wish to point out; but these objections are only offered as my personal opinions, and may have been formed because of a lack of experience in those methods. First, anchoring bridges by means of bars or spans resting in the cavities of adjoining teeth and held in place by fillings, never appealed to me as being a sufficiently strong operation to withstand the strain which a patient will give to a bridge. I have often seen teeth, weakened by approximal cavities, split through the bifurcation of the roots under the strain of ordinary use. How, then, can we expect them to resist the extra strain of a bridge, especially the bicuspids, which I consider the weakest teeth for this purpose? Again, sensitiveness in a large proportion of the teeth will be so great as to prevent excavating the cavity sufficiently to insure a perfect anchorage for the bridge pier. I think the majority of operators will agree with me in this. Another point: at the cervical margin of the filling, where the bar rests, an opportunity exists for food particles to collect which would eventually cause a breaking-down of the enamel.

The doctor calls attention to the contrast between his system and Dr. Brown's. The only difference I note is that Dr. Brown's bridges and bars are all made in one piece, and when once inserted cannot be taken out, while Dr. Flickinger's bars are separate from the bridge and crowns. The bar is first anchored to the teeth, then the bridge put in place and held by small screws into the bar. I see no great advantage in the process except its convenience for removal in case of necessary repairs.

In the case of this single crown, he has the post fitted in the root with amalgam; the band attached to the crown is fitted about the root, then fastened with a screw. If he does not use cement, I think in time the root would decay. If it is cemented before being screwed, what advantage has the process over a crown made in one piece and cemented in the usual manner?

The doctor not being present to advocate his own methods and explain more fully his ideas, I do not wish to take advantage of his absence and criticise the work, but I merely discuss it from my own stand-point.

I approve of saddle-rests in many instances, although much bridge-work is done without any support whatever upon the gum, some dentists doubting its advisability because of the inflammation which might occur to the soft tissues. In extensive bridges where the back teeth have been extracted, leaving no abutment for that end, I use vulcanized rubber under the bridges as saddles and cement them in the mouth. No doubt such a process will be condemned by some, although it would not be if they could have the opportunity which I have had of observing the results. I have never seen any trouble from the gum under the saddles made in this way, some of them having been in use for six or seven years.

I have been experimenting somewhat in the direction of combining the best features of gold bridge-work and those of porcelain work by using the attachments and fittings which, for strength and durability, I believe to be best obtained by the gold-crown process, making the intervening teeth and saddle of porcelain, and connecting into one bridge.

If the experiments and tests of this combination prove fruitful, and a product strong and durable enough to do the work required of it by the patient is secured, I believe the acme of crown- and bridge-work will have been attained.

Dr. Woodward asked me to bring some specimens to substantiate my position. I told him I had nothing at the time which I thought would do justice to the subject, but he glanced about the laboratory and found several things which he persuaded me to bring. I will pass them around, and if they meet with approval, I shall be pleased that I brought them. Two of the models were roughly made. The third case may show the durability of bridge-work. About five years ago a lady came to me for advice. On the left side of her upper jaw she had the central, canine, and second molar; on the right side, the central, lateral, and canine. She wanted a bridge on the left side, but I told her I thought it could not be durable, as her teeth were badly affected with pyorrhœa. She persuaded me to make the bridge, however, and to my surprise it lasted until a few weeks ago. I will now show the model, and I imagine I can hear some present say that the bridge caused the teeth to loosen and come out. But here I would like to emphasize the fact that the three teeth of the right side, which were not connected with the bridge, became loose and uncomfortable, and had to be extracted some weeks before I extracted the bridge.

Another case is that of a gentleman who had a bridge made for his mouth. It was imperfectly made, and soon wore out. This time he wanted something permanent. It is an upper case of eight crowns, anchored to the two canines and left second bicuspid with gold caps. The patient came into my office yesterday at three o'clock, and it is now finished, ready for the mouth. There is one feature about this particular case which I seldom make use of, and whether it is an advantage for strength I am not prepared to say,—that is, the plate rests upon the gum. I made it in this way at the gentleman's request. He speaks a great deal in public, and complained that with his last bridge there was a hissing sound from the top. I usually make these bridges with simply a half round form, of solder, grinding the teeth to fit closely to the gum.

I wish to say with reference to gold showing, that some people like it and some do not; if a patient insists upon having a gold cap, I feel it is my duty to make it. I try to persuade them to have a porcelain facing; but if they like the gold, and their friends like it, it is their business and not mine. With some people, where they would be apt to break the porcelain facing, I often resort to the gold caps in building up the teeth, having the wear come on the ends of the gold caps.

I hesitated about bringing these cases, because I thought this meeting belonged to others, and as I was invited for discussion, it was hardly fair for me to advocate and introduce other methods.

*Dr. Brockway.*—In constructing a bridge for the incisor teeth, with the canines in position and in good condition, what method would Dr. Northrop adopt?

*Dr. Northrop.*—My preference would be to excise the canine teeth and make good solid Richmond crowns, which would give as much strength as any way that could be devised, and show no gold whatever.

*Dr. Brockway.*—Would it not be possible to make skeleton or open-faced crowns?

*Dr. Northrop.*—The only objection I have to that is that bands of considerable width must be had in order to hold the cement. With narrow bands, I have found the cement would dissolve and decay begin. I am opposed to a band, because there is not one tooth in a hundred that I could band which would hold the cement as well as a full cap-fitting. Very often the canines stand parallel with each other. Sometimes the widest point is farther apart than the neck of the tooth, and consequently the band is strained. I have heard of the method of burnishing the band to the neck of a tooth, but it is well known that if burnished to one side it will spring out at the other; so I do not approve of that.

*Dr. Bogue.*—Will Dr. Northrop tell us what cases he considers ought to be cemented firmly?

*Dr. Northrop.*—If I find a case where I can have a good attachment at each end I would cement the bridge, providing the shape of the teeth is such that I can get the bands in place and have them fit perfectly at the neck. The removable bridges I have put in are for very nervous people. I am sometimes afraid to put a permanent case in for fear that future work could not be done; so I put in a bridge that can be taken out, brushed, and cleansed by the patient. In cases where the back teeth have been extracted on both sides evenly I often make a fitting on the last tooth which is in the mouth, and, having a saddle-rest, connect those teeth with a band which does not rest tightly on the teeth or gum, but which can be cleansed. The presence of it is annoying to the patient at first. Sometimes I make what I call double cap-cases, making a gold cap over one or two teeth; then making another cap over that.

*Dr. Bogue.*—Would Dr. Northrop in certain cases cut down a good natural tooth to place a cap for a bridge anchorage?

*Dr. Northrop.*—Yes, in cases where it would be beneficial to do so. I do not think it is a sacrifice of a natural tooth to utilize it to give the patient two teeth. If it is ground down I do not feel that its life is endangered.

*Dr. Bogue.*—How long should a bridge last, the conditions being favorable?

*Dr. Northrop.*—My experience extends back to about 1887. I have pieces made and put in at that time which are as good to-day as when first inserted. Of course, it is possible for a crown to be worn through, or for one side to wear off on account of hard service. I have in mind the case of one gentleman who has had a piece broken three times. It has a good attachment, too. Every time I put it in I build it stronger, and now it is about the thickness of a lead-pencil. There are times when teeth are very loose. I have heard it stated that some teeth were too loose to put a bridge on. I have seen bridges put in upon rather loose teeth, and believe that by the binding of the teeth together their life was longer than if they had been left alone.

*Dr. Bogue.*—Conceding that the durability of loose teeth so treated might be greater, how would it be with teeth that were reasonably firm?

*Dr. Northrop.*—After several years' service, I seldom find that the bridge has been any detriment, or that there is any more movement to the teeth than there was before. I do not say to patients that bridge-work is infallible, or that they will never need to have anything more done. I often tell patients that if they will assume the responsibility, I feel confident that they will have comfortable service enough from the bridge to repay them for the expenditure they make.

*Dr. Peeso.*—I have a case here which I was requested to show as a way not to fit a band. It speaks for itself. Each one has his own ideas in regard to removable and permanent work. Where pieces are extended and a saddle used it strikes me that removable work is an advantage. As to the durability of crown- and bridge-work, if the conditions are favorable, and every part of the work carefully and thoroughly done, it is permanent. If I may be pardoned for referring directly to my own practice, I will say that since the fall of 1888 I have done a great deal of bridge-work, and in all that time there have been but two or three cases which have not proved entirely satisfactory from the first. I have kept track of all the work that I have done, and it is seemingly as good today as when put in the mouth. In many cases where roots are loose, if work is given them to do, they will become strengthened, as is the case with any muscle in the body. As to the pieces that I mentioned as not turning out as they should, in one case failure resulted from disease, the piece having been left out of the mouth for some months, and when it was replaced the conditions were changed and the piece was rendered worthless. I was exonerated from all blame by both the patient and the physician. In the other cases the trouble was but temporary.

*Dr. Elliott.*—I had hoped to have considerable time in which to present several things to the Institute this evening, but I am glad that the time has been so much more profitably occupied. I want to show some of these things to-night, because I cannot remove them from the office.

At a recent meeting I spoke of the use of cataphoresis and the adoption of certain modified forms of apparatus. I have one here on the wall; it can be very readily examined by any one. The only original feature is in the rheostat below, which I had made to order. I use the street-current with a pressure of about one hundred and eighteen volts and the Willms controller. The milliampèremeter is below. The controller has very fine gradations. The rheostat has six steps,—5, 10, 15, 25, 35, 50 volts. I commence with five and have one hundred and ten steps in the controller, which gives me a great deal of gradation. I do not put the negative pole on the wrist now, having done so on several occasions with rather unpleasant results,—red marks and blistering,—but place it in the mouth. It is made of a copper ball placed under the rubber dam. Where we wish to remove a pulp it is necessary to keep the patient under the influence of the current for a long time, and to many people it is difficult to hold the electrode in the mouth for forty or fifty minutes, although I have done so for an hour and a quarter. This apparatus, invented by my son, is something like the gag used when giving gas. It is placed between the jaws, and has a universal motion, so the positive electrode can be placed wherever desired. I very seldom use clamps with the rubber dam, and never really find them necessary. The late Dr. Coffin, of London, introduced as a substitute beads on the silk ligature. The ligature and beads are placed on the teeth, and the rubber jumped over the beads. Using two silk ligatures, one is passed over the tooth and the other through the electrode and tied on, the patient thus being enabled to open and close the mouth.

I have here a vulcanizer of English make, of which the packing is lead and is permanent. I have never had any leakage from it. The regulator is made by the Dental Manufacturing Company of London. The advantage of regulators is very great, and for fifteen years I have used them. The vulcanizer can remain in action for hours, as the regulator keeps the gas absolutely at a fixed point. The advantage of a regulator with a dial over those without is that if one leaves the room he can tell on returning exactly what has taken place. The regulator is first set at sixty pounds pressure, and allowed to run at that pressure for half an hour, when it is turned to ninety pounds for one hour. That requires a slight effort of the memory, and sometimes I have set it at sixty pounds and gone on operating for three hours, and forgotten all about it. That is unpleasant, but has had no disastrous results. This electric regulator has two weights attached to two levers, both supported by the electro-magnet. When the clock makes connection with the current the first weight drops, and in doing so turns up the regulator to ninety pounds. As the regulator requires some force to move it the electro-magnet could not accomplish it alone. When an hour passes the current is again turned on and the second weight is dropped, turning off the gas. In regard to this hand-piece, I do not think it has ever been exhibited. It has a slip-joint, which is not like the S. S. White's, for it can be taken apart without a screw-driver or other tool. The point or bur is held without having to be screwed up, and runs true, but cannot be taken out without slipping back this ring. I have used it for fifteen years, and never knew it to fail. Of course it is not patented. On this same slip-joint I attach my right-angle, direct engine-mallet, and right-angle mallet, all original and tried. Let me here call attention to several kinds of pneumatic mallets, two being made of glass; one being rather curious, inasmuch as the point is attached to the flyer and strikes the gold as a hammer would direct, striking a much heavier blow, not having the inertia of the point-holder to overcome. The glass one has the advantage on account of its lack of friction. I also have a glass Bunsen burner, which answers the purpose better than the metal one, and, as the barrel is always cool, it can be turned up and down by the hand. Here is a soldering appliance for holding work in any position. Here is a compound Bunsen arrangement which can be used at any angle, or as a blow-pipe. Altogether there are some twenty-five appliances here, all original and mostly home-made, which all are invited to examine.

*Dr. Brockway.*—I would move a vote of thanks to Drs. Peeso and Flickinger for their interesting and valuable papers, and for the exhibition of models and finished crowns and bridges with which they have favored us. I would include also those gentlemen who have so kindly come to lead in the discussion.

The motion was carried.

Adjournment.

S. E. Davenport, D.D.S., M.D.S.,
*Editor The New York Institute of Stomatology.*

